# Parodontitis – Therapie einer Volkskrankheit

**DOI:** 10.1007/s00103-021-03373-2

**Published:** 2021-07-08

**Authors:** Bettina Dannewitz, Birte Holtfreter, Peter Eickholz

**Affiliations:** 1 Poliklinik für Parodontologie, Zentrum der Zahn‑, Mund- und Kieferheilkunde, Johann Wolfgang Goethe-Universität Frankfurt am Main, Theodor-Stern-Kai 7, 60596 Frankfurt am Main, Deutschland; 2 Zahnärztliche Gemeinschaftspraxis Dres. Dannewitz & Glass, Weilburg, Deutschland; 3 Poliklinik für Zahnerhaltung, Parodontologie, Endodontologie, Kinderzahnheilkunde und Präventive Zahnheilkunde, Zentrum für Zahn‑, Mund- und Kieferheilkunde, Universitätsmedizin Greifswald, Greifswald, Deutschland

**Keywords:** Parodontitis, Parodontitistherapie, Diagnostik, Leitlinien, Periodontitis, Periodontal therapy, Diagnostics, Guideline

## Abstract

Parodontitis ist eine chronisch entzündliche nichtübertragbare Erkrankung, die alle Anteile des Zahnhalteapparates (Parodonts) betrifft und dort weitgehend irreversible Schäden verursacht. Schätzungen legen nahe, dass in Deutschland ca. 10 Mio. Menschen an einer schweren Parodontitis erkrankt sind. Parodontitis zeigt über viele Jahre zumeist wenige oder nur milde Symptome, die von den Patienten oft nicht wahrgenommen oder richtig eingeordnet werden. Fehlendes Bewusstsein kann dazu führen, dass zahnärztliche Behandlung erst in einem fortgeschrittenen Erkrankungsverlauf in Anspruch genommen wird, wenn umfangreiche Therapiemaßnahmen notwendig geworden sind und sich die Prognose für den Erhalt der Zähne verschlechtert hat. Der parodontale Screeningindex (PSI) ist ein einfaches und schnelles Instrument, mit dem die Notwendigkeit weiterführender diagnostischer Maßnahmen beurteilt werden kann. Der Index wird mittlerweile bei vielen Patienten durchgeführt. Trotzdem bleiben die Versorgungszahlen niedrig und hinter dem zurück, was für das Absenken der bestehenden Parodontitislast notwendig wäre. Jede Zahnarztpraxis muss in der Lage sein, Parodontitistherapie umzusetzen. Fachzahnärzte oder Spezialisten können die allgemeinzahnärztlichen Kollegen wesentlich bei der Behandlung von schweren Formen von Parodontitis unterstützen. Dazu ist eine Aufwertung des Faches in der universitären Ausbildung erforderlich, aber auch die zunehmende postgraduale Ausdifferenzierung von Spezialisten oder Fachzahnärzten für Parodontologie. Die neuen Behandlungsrichtlinien für die Parodontaltherapie (PAR-Therapie) erlauben die Versorgung der parodontal erkrankten Patienten auf Basis international anerkannter wissenschaftlicher Standards und verbessern damit die Rahmenbedingungen für die Parodontitistherapie in der zahnärztlichen Praxis.

## Abstract (en)

Periodontitis is a chronic inflammatory noncommunicable disease that affects all parts of the periodontium and causes irreversible damage. It is estimated that around 10 million people in Germany suffer from severe forms of periodontitis. The disease usually shows few or only mild symptoms over many years, which are often not perceived or correctly classified by the patient. A lack of awareness could lead to dental treatment being sought in an advanced stage of the disease when extensive therapeutic measures have become necessary and the prognosis for tooth retention has worsened. The periodontal screening index (PSI) is a simple and rapid tool that is used to assess the level of further examination needed. The index is now carried out on many patients. However, the number of treatments remain low and behind what is necessary to reduce the existing burden of periodontitis. Every dental practice must be able to implement periodontal therapy in their clinical setting. Periodontal specialists can support general dentists significantly, especially in the treatment of severe forms of periodontitis. This requires an upgrading of the subject in university education, but also an increasing postgraduate differentiation of specialized dentists for periodontology. The new treatment guidelines for PAR (periodontal) therapy allow periodontal treatment on the basis of internationally recognized scientific standards and thus improve the framework conditions for therapy in the dental practice.

## Einleitung

**Figure 1:**
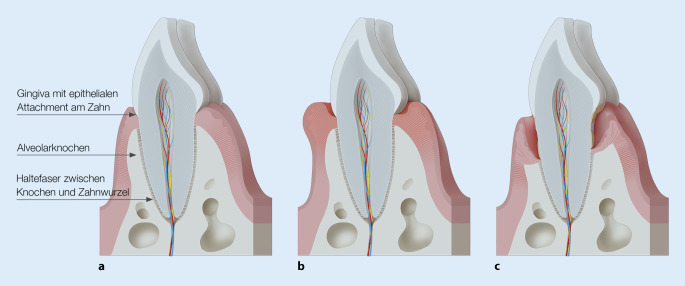

Der Zahnhalteapparat (Parodont) besteht aus verschiedenen Gewebetypen (Epithel, Bindegewebe, Zement und Knochen), die nicht nur für die Verankerung des Zahnes im Kieferknochen sorgen, sondern auch einen dichten Verschluss um den Zahn ausbilden, um dort das Eindringen von oralen Mikroorganismen in den Körper zu verhindern (Abb. 1).

Parodontalerkrankungen zählen zu den häufigsten Erkrankungen weltweit [1]. Neben Parodontitis und Gingivitis (Zahnfleischentzündung) gibt es noch weitere Erkrankungen und Veränderungen, die das Parodont betreffen können [2]. Von ihnen hat Parodontitis allerdings die stärksten Auswirkungen auf die (orale) Gesundheit von Erwachsenen und das Gesundheitswesen (gesundheitsökonomische Aspekte). Die Prävalenz der Erkrankung nimmt mit steigendem Alter allmählich zu. Dabei haben die meisten Betroffenen einen leichten bis moderaten Krankheitsverlauf. Schwere Formen von Parodontitis treten vor allem im höheren Erwachsenenalter und bei Senioren auf. In der Global Burden of Disease Study aus dem Jahr 2015 wurde die Prävalenz für schwere Parodontitis weltweit auf 7,4 % geschätzt [3]. Schätzungen auf Basis der Daten der 5. Deutschen Mundgesundheitsstudie (DMS V) legen nahe, dass in Deutschland ca. 10 Mio. Menschen an einer schweren Parodontitis erkrankt sind [4]. Jährlich werden dagegen nur etwa 1 Mio. systematische Parodontalbehandlungen mit den gesetzlichen Krankenkassen (GKV) abgerechnet [5]. In diesem Artikel soll ein Überblick über etablierte diagnostische Verfahren und die Therapie von Parodontitis gegeben und mögliche Gründe für die Diskrepanz zwischen hohem Versorgungsbedarf und unzureichender Inanspruchnahme bzw. Behandlungszahlen dargestellt werden.

### Parodontitis – warum verlieren Zähne ihren Halt?

Parodontitis ist eine chronisch entzündliche nichtübertragbare Erkrankung, die alle Anteile des Zahnhalteapparates betrifft und weitgehend irreversible Schäden des Parodonts verursacht (Abb. 1). Bei einer Gingivitis dagegen bleibt die Entzündung auf das Zahnfleisch (Gingiva) beschränkt, die dabei auftretenden klinischen und histologischen Veränderungen sind im Gegensatz zur Parodontitis reversibel. Gingivitis und Parodontitis geht immer die Akkumulation von Biofilm (Schicht aus einer Mischpopulation von Mikroorganismen auf Oberflächen) im Grenzbereich von Zahn und Gingiva voraus. In der Mundhöhle gibt es eine Reihe ganz unterschiedlicher Habitate für die oralen Mikroorganismen, dabei bieten Zähne mit ihrer nicht abschilfernden Oberfläche sehr günstige Bedingungen für die Anlagerung und langfristige Kolonisation von Bakterien. Zudem enthalten der Speichel und die gingivale Sulkusflüssigkeit (Flüssigkeit in der Zahnfleischtasche) Nährstoffe für das bakterielle Wachstum, aber auch antibakteriell wirksame Komponenten [6].

Durch die Unterschiede in den ökologischen Bedingungen auf den supra- und subgingivalen Zahnoberflächen bilden sich in den Biofilmen dieser ökologischen Nischen spezifisch angepasste, strukturell und funktionell organisierte mikrobielle Gemeinschaften aus [7]. Das komplexe Gleichgewicht zwischen den verschiedenen bakteriellen Spezies beeinflusst dabei maßgeblich die Entstehung von oralen Erkrankungen wie Karies, Gingivitis und Parodontitis. Bei parodontaler Gesundheit besteht eine Symbiose zwischen dem Biofilm und einer angemessenen immuninflammatorischen Wirtsantwort. Selbst ein klinisch gesundes Parodont zeigt histologisch immer eine begrenzte Infiltration von Entzündungszellen im Bereich des epithelialen Attachments der Gingiva.

**Figure 2:**
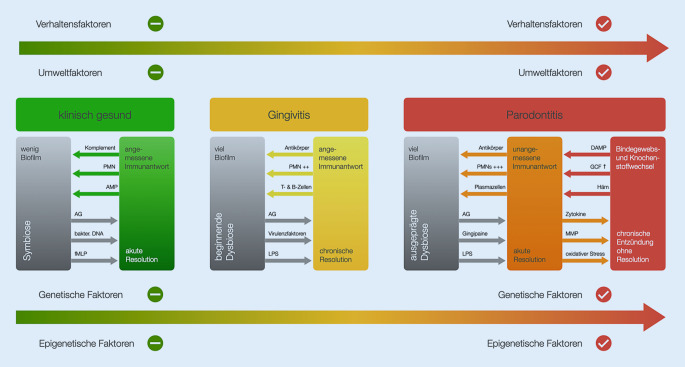

Die bakterielle Kolonisation der Zahnoberflächen induziert zunächst nur eine Entzündungsreaktion in der Gingiva, die durch das Immunsystem moduliert wird und über lange Zeit persistieren kann. Verschiedene Einflüsse können aber eine Störung des Ökosystems im Mund bewirken und durch das Überwachsen spezifischer, meist gramnegativer Pathobionten (Symbionten, die unter bestimmten Bedingungen pathologisch werden) eine Veränderung des subgingivalen Biofilms in Richtung einer proinflammatorisch wirkenden Dysbiose (Ungleichgewicht der Mikroflora) induzieren [8–10]. Durch diese Dysbiose kann es bei dafür anfälligen Individuen zu Parodontitis kommen, die mit einer Fehlsteuerung der inflammatorischen Antwort einhergeht und bei der es zu einem Abbau von Bindegewebe und Alveolarknochen kommt (Abb. 2). Man geht davon aus, dass Parodontitis immer eine Gingivitis vorausgeht [11]. Allerdings entwickelt sich nicht aus jeder Gingivitis eine Parodontitis und es ist bisher nicht möglich, diese Fälle zu identifizieren, bevor die Schäden am Parodont röntgenologisch und klinisch messbar werden [12].

Parodontitis ist eine multifaktorielle Erkrankung. Der Verlauf und die Schwere der Erkrankung werden neben der bakteriellen Ätiologie durch eine Reihe weiterer Faktoren bestimmt, die das dynamische Gleichgewicht der Interaktionen zwischen Mikroorganismen und Wirt beeinflussen können, unter anderem metabolisch schlecht eingestellter Diabetes mellitus, Nikotinkonsum, Stress, genetische und lokale Faktoren, wie z. B. Zahnengstand [12, 13]. Wie bei anderen chronischen Erkrankungen besteht ein deutlicher Zusammenhang zwischen Parodontitis und sozioökonomischen Variablen sowie dem Verhalten einschließlich der Exposition von Risikofaktoren und den individuellen Mundhygienemaßnahmen [14].

### Parodontitis – ein unerkanntes oder übersehenes Problem?

Der Verlauf von Parodontitis ist zumeist langsam und schmerzlos, leichte und moderate Formen von Parodontitis zeigen daher über viele Jahre zumeist wenige oder nur milde Symptome. Veränderungen der Gingiva (Zahnfleischbluten, Rötung und Schwellung) sind oft die ersten und einzigen Anzeichen, die von den Patienten oft nicht wahrgenommen oder richtig eingeordnet werden, bis es dann zu einer Lockerung der Zähne kommt. Gingivitis und frühe Stadien der Parodontitis sind für Betroffene nicht zu unterscheiden. Fortgeschrittene Formen von Parodontitis sind durch eine deutliche Zerstörung des parodontalen Ligaments und des Knochens gekennzeichnet, die zu einer merklichen Lockerung der Zähne, Zahnwanderung, einem Rückgang der Gingiva mit „schwarzen Dreiecken“ in den Zahnzwischenräumen und freiliegenden Zahnhälsen führen. Trotzdem wird Parodontitis oft als „stille“ Erkrankung beschrieben [15].

Bei einer Untersuchung wurde die Selbsteinschätzung der parodontalen Situation mit dem klinischen Befund verglichen. Dabei zeigte sich, dass etwa 75 % der Studienteilnehmer von einer moderaten oder schweren Form von Parodontitis betroffen waren, aber 62–86 % dieser beiden Gruppen nicht der Meinung waren, an Parodontitis erkrankt zu sein [16]. Bei einer telefonischen Befragung in Deutschland wurde deutlich, dass in der Bevölkerung erhebliche Defizite beim Wissen darüber, was Parodontitis ist und welche Konsequenzen die Erkrankung haben kann, bestehen, aber auch welche Risikofaktoren mit der Erkrankung assoziiert sind und welche präventiven Maßnahmen effektiv sein können [17]. Das fehlende Bewusstsein für die eigene Erkrankung kann dazu führen, dass zahnärztliche Behandlung erst dann in Anspruch genommen wird, wenn es bereits zu massiven Gewebeverlusten gekommen ist. Dadurch werden umfangreiche Therapiemaßnahmen notwendig und die Prognose für den Erhalt der Zähne verschlechtert sich meist deutlich.

Wenn in Deutschland ca. 10 Mio. Menschen an schwerer Parodontitis erkrankt sind [4], aber jährlich nur etwa 1 Mio. systematische Parodontalbehandlungen mit den gesetzlichen Krankenkassen abgerechnet werden [5], würde es theoretisch 10 Jahre benötigen, um diese Erkrankungslast zu therapieren. Darüber hinaus liegt bei 1 Neuerkrankung an schwerer Parodontitis unter 142 Personen pro Jahr die jährliche Neuerkrankungsrate bei einer Bevölkerung von 80 Mio. bei etwa 500.000 [18], was die Lage verschärft. Diese Situation kann durchaus als Unterversorgung bewertet werden, für die es unterschiedliche Gründe geben kann.

Wie sieht es mit der Ausbildung der Zahnärzte hinsichtlich parodontaler Erkrankungen aus? Die noch gültige Approbationsordnung für Zahnärzte sieht im Rahmen der Zahnärztlichen Prüfung in der Fächergruppe Zahnerhaltungskunde eine eigene Prüfung in Parodontologie vor. Das alleine stellt aber noch nicht sicher, dass in der zahnmedizinischen Ausbildung genug Wert auf parodontale Aspekte gelegt wird. An den deutschen Universitätseinrichtungen, die Zahnmedizin ausbilden, sind regelhaft Professoren für Zahnerhaltungskunde, zahnärztliche Prothetik, Kieferorthopädie sowie Mund‑, Kiefer‑, Gesichtschirurgie berufen. Professuren für Parodontologie sind eher die Ausnahme [19]. Dies ist ein Indiz dafür, welch geringe Bedeutung dem Fach beigemessen wird. Ohne Professur hat es eine Fachdisziplin schwer, nachhaltige Bedeutung in der Ausbildung zu erlangen.

Deutschland weist im Vergleich zum Ausland mit rund 87 Zahnärzten pro 100.000 Einwohner eine besonders hohe Dichte an Zahnärzten auf (Quelle: statista, www.de.statista.com). Jedoch ist im Gegensatz zu anderen Industrienationen die Weiterbildung von Fachärzten hierzulande noch nicht weit entwickelt. Anders als in den Fächern Kieferorthopädie und zahnärztliche Chirurgie gibt es nicht in allen Zahnärztekammern eine Möglichkeit zur Weiterbildung zum/zur Fachzahnarzt*ärztin für Parodontologie. Lediglich die Zahnärztekammer Westfalen-Lippe sieht eine solche Weiterbildung vor. Seit Jahrzehnten wehren sich andere Zahnärztekammern gegen eine fachzahnärztliche Weiterbildung im Bereich Parodontologie und begründen dies mit dem Argument, Parodontitis könnte auch durch nicht weitergebildete Zahnärzte behandelt werden. Dies trifft allerdings auch für kieferorthopädische und oralchirurgische Leistungen zu, die jeder Zahnarzt auch ohne Zusatzqualifikation erbringen kann. Ein wichtiger Grund für die fehlende Ausdifferenzierung durch die Zahnärztekammern ist vermutlich auch, dass die Verhandlungsposition der Kassenzahnärztlichen Vereinigung gegenüber den Kostenträgern durch die Aufspaltung der Zahnärzteschaft in verschiedene Fachgruppen nicht geschwächt werden soll.

Möglicherweise können nicht weitergebildete Zahnärzte leichte und moderate Formen von Parodontitis tatsächlich gut behandeln, aber offenbar erfolgt bisher keine ausreichende Umsetzung. Bei der bestehenden Prävalenz der Parodontitis muss parodontale Prävention und Therapie notwendigerweise Teil des Therapiekonzeptes einer jeden Zahnarztpraxis sein. Nur mit Fachzahnärzten sind die hohen Zahlen nicht zu bewältigen, aber Fachzahnärzte könnten die allgemeinzahnärztlichen Kollegen wesentlich unterstützen.

Die Deutsche Gesellschaft für Parodontologie (DG PARO) hat deshalb vor knapp 30 Jahren eine der Fachzahnarztausbildung analoge Ausbildung zu Spezialisten für Parodontologie ins Leben gerufen. Die Ausbildung zum DG PARO-Spezialisten für Parodontologie® ist mit der Ausbildung zum Fachzahnarzt für Parodontologie identisch und umfasst eine Ausbildung von ca. 5000 h. In Deutschland gibt es aktuell 244 solche DG PARO-Spezialisten für Parodontologie (Quelle: Deutsche Gesellschaft für Parodontologie e. V., Stand Ende 2020). Deutlich stärker steigen dagegen die Zahlen von Absolventen postgradualer Masterprogramme in diesem Fachgebiet. Die Ausbildung mit insgesamt ca. 3200 h erfolgt zumeist berufsbegleitend und hat eine stärkere theoretische Ausrichtung als die des Spezialisten und des Fachzahnarztes.

Weitere mögliche Erklärungen für die geringen Behandlungszahlen sind die bisher zumindest zum Teil inkonsistenten Richtlinien, die die systematische Therapie von Parodontopathien im Rahmen der GKV regeln, sowie die nicht ausreichende Honorierung dieser Therapie. Am 17.12.2020 hat der Gemeinsame Bundesausschuss (G-BA) nach einem fast 10 Jahre währenden Prozess eine neue Behandlungsrichtlinie beschlossen, die parodontale Therapie nach modernen evidenzbasierten Standards erlaubt.^1^

### Parodontitis – mehr als lockere Zähne

Unbehandelt oder unzureichend therapiert führt Parodontitis zu einer Zerstörung der zahntragenden Gewebe und letztendlich dem Verlust von Zähnen. Die Erkrankung ist eine der Hauptursachen für Zahnverlust bei Erwachsenen weltweit [20, 21] und hat damit einen negativen Einfluss auf Kaufunktion, orale Ästhetik und Lebensqualität der Betroffenen. Eine systematische Therapie von Parodontitis kann dagegen die Lebensqualität der Betroffenen signifikant verbessern [22, 23].

Durch die Erkrankung selbst und den Ersatz von fehlenden Zähnen trägt Parodontitis in erheblichem Maße zu den Kosten bei, die direkt oder indirekt durch Zahnerkrankungen verursacht werden (jährlich etwa 544 Mrd. USD weltweit, davon entfallen auf Parodontitis Kosten von schätzungsweise 79 Mrd. USD [24, 25]), und verstärkt soziale Ungleichheit [1].

Außer den lokalen Schäden am Parodont kann Parodontitis auch Auswirkungen auf den gesamten Körper haben und steht in Zusammenhang mit einer Vielzahl von systemischen Erkrankungen, u. a. Diabetes mellitus [26], kardiovaskulären Erkrankungen [27], Schwangerschaftskomplikationen [28] und Demenz [29]. Ergebnisse einer aktuellen Studie zeigen zudem, dass Menschen mit Parodontitis, die an COVID-19 erkranken, ein signifikant höheres Risiko für schwere Komplikationen (Einweisung auf Intensivstationen, Beatmung und Tod) im Verlauf der Infektion haben [30]. Dabei teilen viele dieser Erkrankungen gemeinsame Risikofaktoren, die auch für die Ätiologie von Parodontitis relevant sind. Schwere Parodontitis verursacht selbst und insbesondere im Zusammenhang mit systemischen Erkrankungen mehr Jahre an Arbeitsunfähigkeit als jede andere Erkrankung des Menschen [31].

Auch wenn zahlreiche Wechselwirkungen mit systemischen Erkrankungen in der Literatur beschrieben werden, ist der bidirektionale Zusammenhang zwischen Diabetes und Parodontitis am besten belegt [26]. Eine nachhaltige und systematische Therapie von Parodontitis könnte insbesondere bei Diabetespatienten einen positiven Beitrag zum Management ihrer Erkrankung leisten und damit Gesundheitskosten reduzieren. Dafür ist aber auch die stärkere Interaktion zwischen Zahn- und Humanmedizinern bei der Versorgung gemeinsamer Patienten notwendig.

### Parodontitis – vom Screening bis zur Diagnose

Parodontitis ist gekennzeichnet durch eine fortschreitende Destruktion des Zahnhalteapparats. Primäres Merkmal ist der Verlust der parodontalen Gewebe, welcher sich klinisch durch Attachmentverluste, Ausbildung parodontaler Taschen, gingivale Blutung und radiologisch nachweisbaren Knochenabbau manifestiert [32]. Frühe Stadien der Parodontitis können durch Patienten aber zumeist nicht von einer Gingivitis unterschieden werden. Wenn die Symptome für den Patienten deutlicher wahrnehmbar werden (freiliegende Zahnhälse, bewegliche Zähne), ist zumeist sehr viel Parodont verloren gegangen und die Therapie damit aufwendig und weniger vorhersagbar. Die Unterscheidung zwischen Gingivitis und Parodontitis muss durch den/die Zahnarzt*ärztin erfolgen. Zu diesem Zweck wird der sogenannte parodontale Screeningindex (PSI) erhoben. Dies dauert 2–3 min [33] und wird von der GKV alle 2 Jahre einmal bezahlt (BEMA^2^-Position 04). Der Code 0 markiert parodontale Gesundheit, die Codes 1 und 2 Gingivitis und die Codes 3 und 4 weisen auf eine parodontale Erkrankung hin. Welcher Art die Erkrankung ist, muss durch weiterführende Diagnostik (Erhebung des sog. Parodontalstatus) geklärt werden. Die Bestimmung des PSI ist seit 2004 Bestandteil der vertragszahnärztlichen Versorgung. Seit seiner Einführung in den BEMA kann ein jährlicher Anstieg dieser Position um ca. 3 % beobachtet werden.

Um beurteilen zu können, bei wie vielen GKV-Patienten der PSI erhoben wurde, ist es sinnvoll, aufgrund der Abrechnungsvorgaben einen Zeitraum von 2 Jahren zu betrachten. In den Jahren 2018 und 2019 wurde die BEMA-Position 04 in etwa 34,6 Mio. Fällen abgerechnet. In diesem Zeitraum waren ca. 72,5 Mio. Menschen gesetzlich krankenversichert. Damit wurde der PSI bei etwa 48 % der GKV-Patienten innerhalb von 2 Jahren erhoben. Legt man die Zahlen der DMS V für die Häufigkeit schwerer Parodontalerkrankungen (CPI-Grad 4 in Analogie zum PSI-Code 4) zugrunde (jüngere Erwachsene: 10,4 %, jüngere Senioren: 24,6 %) und schätzt konservativ mit einer mittleren Prävalenz von 17,5 % für die gesamte Bevölkerung, hätten 2018/2019 theoretisch etwa 5,3 Mio. Patienten, bei denen der PSI ermittelt wurde, einen Code 4 haben müssen. Damit wäre die Indikation für die Erhebung eines kompletten Parodontalstatus als Basis einer eventuell notwendigen Parodontitistherapie gegeben. Im gleichen Zeitraum wurden allerdings nur etwa 2 Mio. systematische Parodontalbehandlungen durchgeführt. Auch wenn sich die Erhebung des PSI zunehmend in den zahnärztlichen Praxen etabliert, bleibt die Frage, ob aus dem Screeningbefund tatsächlich die richtigen Schlussfolgerungen und therapeutischen Konsequenzen gezogen werden.

**Figure 3:**
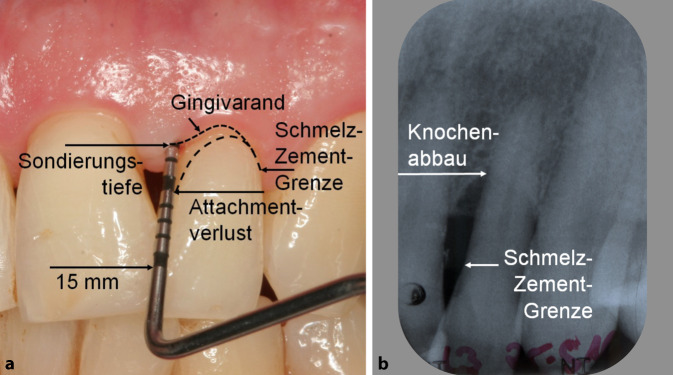

Um die Schwelle für das Parodontitisscreening zu senken, hat die DG PARO einen Selbsttest entwickelt, mit dessen Hilfe Patienten über eine Smartphone-App^3^ ihr individuelles Erkrankungsrisiko abschätzen können. Die Abklärung des Verdachts auf Parodontitis muss dann aber doch beim/bei der Zahnarzt*ärztin erfolgen (Abb. 3).

Bei der klinischen Untersuchung kann die parodontale Zerstörung als Attachmentverlust oder Knochenabbau bestimmt und ihr Ausmaß entsprechend gemessen werden (Abb. 3). Deshalb kommt Röntgenbildern bei der Diagnostik der Parodontitis ebenfalls eine große Bedeutung zu. Gerade die Art und Verteilung der Zerstörung des parodontalen Knochens (z. B. Knochentaschen) kann auf Röntgenbildern besser beurteilt werden. Dentale Biofilme bzw. orale Bakterien spielen zwar eine zentrale Rolle in der Parodontitispathogenese und von etwa 700 in der Mundhöhle nachweisbaren Bakterienstämmen werden bei Patienten mit Parodontitis bestimmte Gruppen häufiger detektiert [34], dennoch kann von diesen nur ein Bruchteil routinemäßig mit kommerziellen Tests nachgewiesen werden. Die pathogene Relevanz der anderen Bakterien ist ungeklärt. Die Auswahl keimspezifischer Antibiotika auf Basis von mikrobiologischen Testergebnissen erscheint daher nicht sinnvoll, auch weil sich keine therapeutische Konsequenz ableiten lässt [35].

**Figure 4:**
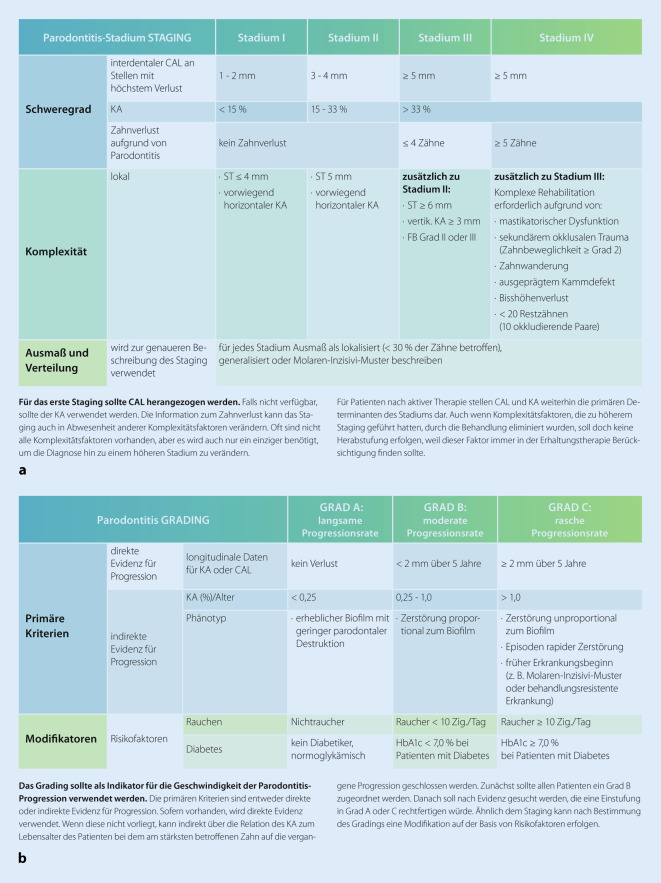

Im Jahr 2018 wurde eine neue Klassifikation der parodontalen Erkrankungen und Zustände veröffentlicht (Abb. 4). Parodontitis wird eingeteilt nach Schweregrad (das sogenannte Staging, 4 Stadien: I, II, III, IV), der sich primär nach Attachmentverlust/Knochenabbau und Zahnverlust richtet, Ausdehnung (lokalisiert, generalisiert, Molaren-Inzisiven-Muster) und Progressionsrate (3 Grade: A, B, C). Das Grading wird in der Praxis zumeist indirekt über den prozentualen Knochenabbau in Bezug zum Lebensalter und das Vorliegen von Modifikatoren (Rauchen und den HbA1c-Wert bei Diabetikern) bestimmt [2, 32, 36].

Die neue Klassifikation wurde auf Ergebnisse der regionalen populationsbasierten Studie SHIP-Trend in Vorpommern [37, 38] angewendet. Für SHIP-Trend‑0 wurde eine stratifizierte Zufallsstichprobe mit 10.000 Erwachsenen im Alter von 20 bis 79 Jahren gezogen, die Nettostichprobe umfasste 8826 Personen, von denen 4420 Teilnehmer zwischen 2008 und 2012 untersucht und 3234 in die Analysen eingeschlossen werden konnten (1650 Frauen und 1584 Männer; [39]). Das Durchschnittsalter lag bei 48,3 ± 14,4 Jahren (70 % älter als 40 Jahre). Es erfolgte eine Einteilung der Teilnehmer in solche mit parodontaler Gesundheit, lokalisierter Gingivitis oder generalisierter Gingivitis, Patienten mit reduziertem, aber gesundem Parodont, Parodontitispatienten mit gingivaler Entzündung sowie Parodontitispatienten der Stadien I bis IV [25, 36]. Probanden, die im Interview eine Parodontitisbehandlung angegeben hatten, wurden als Parodontitispatienten definiert und entsprechend in das Staging einbezogen. Diese differenzierte Einteilung in Schweregrade geht über die Beurteilung der parodontalen Situation in der DMS V hinaus und erlaubt damit auch eine bessere Einschätzung des tatsächlichen Behandlungsbedarfes in Deutschland. Nur 4,7 % der Teilnehmer waren parodontal gesund, 6,9 % hatten eine Gingivitis (lokalisiert oder generalisiert), 23,7 % der Patienten hatten nach eigenen Angaben eine Parodontitistherapie durchlaufen und zeigten ein reduziertes, aber gesundes Parodont bzw. mit lokalisierten Entzündungen, 67 % waren an einer Parodontitis erkrankt. Auf der Grundlage der aktuellen Klassifikation wurden 8,4 % den Stadien I und II und 56,4 % den Stadien III und IV zugeordnet (Tab. 1).

**Table 1:** 

		Gesamt % (SE)	Frauen % (SE)	Männer % (SE)
Parodontal gesund	–	4,7 (0,4)	5,6 (0,7)	3,8 (0,5)
Lokalisierte Gingivitis	–	4,1 (0,4)	4,1 (0,5)	4,0 (0,5)
Generalisierte Gingivitis	–	2,8 (0,3)	2,9 (0,4)	2,7 (0,4)
Patienten mit reduziertem, aber gesundem Parodont (Patienten nach PAR-Therapie)	–	14,0 (0,6)	15,9 (0,9)	12,3 (0,9)
Parodontitispatienten mit gingivaler Entzündung (Patienten nach PAR-Therapie)	–	9,7 (0,5)	10,8 (0,8)	8,6 (0,7)
Parodontitis Stadium I	Insgesamt 67,0	1,4 (0,2)	1,4 (0,4)	1,3 (0,3)
Parodontitis Stadium II		7,0 (0,5)	5,4 (0,6)	8,6 (0,7)
Parodontitis Stadium III		27,7 (0,8)	25,4 (1,0)	29,9 (1,1)
Parodontitis Stadium IV		28,7 (0,7)	28,5 (1,0)	28,8 (1,0)

Analyse mit Berücksichtigung der Anzahl der fehlenden Zähne, die Gründe für die Zahnextraktion konnten nicht eruiert werden. Aufgrund des komplexen Studiendesigns wurden die Standardfehler unter Berücksichtigung der Stichprobengewichte und der Stratifizierungsvariablen berechnet

*PAR-Therapie* Parodontaltherapie, *SE* Standardfehler

### Parodontitistherapie – schrittweise zum Ziel

**Figure 5:**
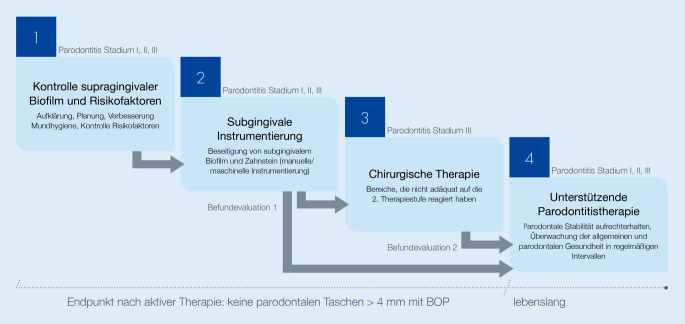

Notwendige Voraussetzung für die Entstehung von Parodontitis ist die durch einen dysbiotischen Biofilm verursachte entzündliche Zerstörung des Zahnhalteapparates. Ein Großteil der Therapiemaßnahmen ist deshalb auf die Kontrolle des dentalen Biofilms gerichtet. Die European Federation of Periodontology (EFP) hat 2020 eine klinische Leitlinie für die Therapie von Parodontitis der Stadien I, II und III veröffentlicht [25], die von der DG PARO 2021 implementiert wurde (Abb. 5). Danach erfolgt die Behandlung der Parodontitis stufenweise (Abb. 5). In der ersten Stufe werden die Patienten zu effektiver Kontrolle der bakteriellen Zahnbeläge geschult, Beläge und lokale Reizfaktoren wurden zusätzlich professionell beseitigt. Risikofaktoren für Parodontitis, wie insbesondere das Rauchen, werden thematisiert und im Idealfall reduziert. In Stufe 1 geht es um die schwierige Aufgabe, das Verhalten der Patienten dauerhaft zu beeinflussen, zu ändern und damit die Grundlage für die weitere Therapie zu legen.

In Stufe 2 der Therapie erfolgt die Beseitigung des subgingivalen Biofilms und Zahnsteins durch nichtchirurgische subgingivale Instrumentierung (SI). Für bestimmte Patientengruppen mit nachgewiesener rascher Progression (z. B. generalisierte Stadien III/IV der Parodontitis bei jungen Erwachsenen) kann die Verwendung systemischer Antibiotika erwogen werden. 3–6 Monate nach SI werden die klinischen Befunde überprüft (Befundevaluation 1). In den meisten Fällen (Parodontitis der Stadien I und II) sollten nach Therapiestufe 2 gesunde Verhältnisse vorliegen (ST ≤ 3 mm und 4 mm ohne Bluten auf Sondieren). Resttaschen mit ST von 4 mm bzw. 5 mm können durch erneute SI in der unterstützenden Parodontitistherapie (UPT) kontrolliert werden [25, 40]. Bei Parodontitis der Stadien III und IV bleiben nach Therapiestufe 1 und 2 oft an einzelnen Zähnen Resttaschen mit ST ≥ 6 mm zurück, die in Stufe 3 chirurgisch therapiert werden.

Bei parodontalchirurgischen Verfahren geht es im Wesentlichen darum, Biofilm und Zahnstein von den subgingival gelegenen Zahnflächen nach Freilegung durch die Mobilisation eines Zahnfleischlappens unter Sicht zu entfernen (Zugangslappenoperation, offenes Vorgehen). Bei bestimmten Defekten (Knochentaschen, Furkationsbefall Grad II) besteht die Möglichkeit, den durch Parodontitis zerstörten Zahnhalteapparat zu regenerieren [41, 42]. Parodontalchirurgische Verfahren sind wirkungsvoll, aber häufig auch technisch anspruchsvoll. Das gilt insbesondere für die regenerative Parodontalchirurgie [43, 44]. Deshalb sollten diese Interventionen durch spezifisch fort- oder weitergebildete Zahnärzte durchgeführt werden [25]. Das Deutsche Zahnheilkundegesetz sieht allerdings keine qualifikationsspezifischen Einschränkungen der zahnärztlichen Berufsausübung vor, d. h., mit der Approbation darf der/die Zahnarzt*ärztin das gesamte Spektrum der Zahnheilkunde ausüben, aber es muss auch klar sein, dass z. B. der Nationale Kompetenzbasierte Lernzielkatalog Zahnmedizin [45] keine Ausbildung der Zahnmedizinstudierenden in Parodontalchirurgie auf dem Niveau der Handlungskompetenz vorsieht. Die Kompetenz auf diesem Feld muss postgradual erworben werden. Die aktuelle Leitlinie fordert allerdings, dass der Zugang zu dieser Versorgung für die Patienten verbessert werden soll. Eine Möglichkeit dazu wäre die bundesweite Einführung eines Fachzahnarztes für Parodontologie.

3–6 Monate nach Abschluss der parodontalchirurgischen Therapie erfolgt ebenfalls eine Überprüfung des Behandlungsergebnisses (Befundevaluation 2). Allerdings ist die Behandlung nun noch nicht abgeschlossen. Kehren die Ursachen der entzündlichen Zerstörung des Parodonts (bakterielle Zahnbeläge, dysbiotischer Biofilm) zurück, wird es auch wieder zu entzündlicher Zerstörung kommen (Rezidiv). Individuelle häusliche Biofilmkontrolle erfordert ein gewisses Maß an Disziplin. Deshalb werden Parodontitispatienten, deren erhöhtes Risiko, an Parodontitis zu erkranken, sich bereits unverkennbar manifestiert hat, schließlich in ein Nachsorgeprogramm UPT eingeschleust, das, je nach individuellem Risiko, 1–4 Termine pro Jahr umfasst. Im Rahmen der UPT werden in jeder Sitzung die individuelle Biofilmkontrolle beurteilt und nachgeschult, die parodontalen Befunde überprüft und bei Bedarf erneut erkrankte Taschen in einem frühen Stadium mit einfachen Mitteln gereinigt. Es konnte gezeigt werden, dass Parodontitispatienten, die regelmäßig an der UPT teilnehmen, langfristig weniger Zähne verlieren als solche, die nur unregelmäßig teilnehmen [46, 47].

Die neue Richtlinie zur systematischen Behandlung von Parodontitis und anderer Parodontalerkrankungen (PAR-Richtlinie), die der Gemeinsame Bundesausschusses am 17.12.2020 beschlossen hat, entspricht im Wesentlichen dem Behandlungsprotokoll der EFP-/DG PARO-Leitlinie. Mit dieser Behandlungsrichtlinie, die die parodontale Therapie der gesetzlich Versicherten regelt, werden Inkonsistenzen der bisher gültigen Richtlinie beseitigt. Ein besonderes Augenmerk wurde dabei auf das zahnärztliche Gespräch gelegt („sprechende Zahnmedizin“), das unverzichtbar ist, wenn eine Verhaltensbeeinflussung der Patienten erreicht werden soll. Außerdem wurde am Übergang von der nichtchirurgischen Therapie (Stufe 2) zur chirurgischen Therapie (Stufe 3) die Überprüfung des parodontalen Befunds eingeführt, die erst die Entscheidungsgrundlage dafür liefert, ob chirurgisch weitertherapiert werden muss. Schließlich wurde die UPT, also das Element, das eine langfristige Stabilität des Behandlungsergebnisses erst ermöglicht, zumindest für 2 Jahre in die vertragszahnärztliche Versorgung eingeführt. Da sich die Häufigkeit der UPT-Sitzungen nach der Progressionsrate (Grad A, B, C) richtet, bekommt die vertragszahnärztliche Versorgung ein Element individualisierter Zahnmedizin. So erlaubt die neue PAR-Richtlinie im Wesentlichen die Umsetzung parodontaler Therapie auf der Basis international anerkannter wissenschaftlicher Standards [25].

## Fazit

Eines der wichtigsten Ziele zahnmedizinischer Versorgung muss es sein, die hohe Parodontitislast in Deutschland zu senken. Dazu ist eine umfassende Information der Menschen über diese Erkrankung, ihre Risikofaktoren, die diagnostischen und therapeutischen Möglichkeiten essenziell. Bei der hohen Parodontitisprävalenz muss parodontale Prävention und Therapie notwendigerweise im Behandlungsspektrum jeder Zahnarztpraxis sein. Fachzahnärzte oder Spezialisten könnten die allgemeinzahnärztlichen Kollegen aber wesentlich unterstützen, vor allem bei der Behandlung schwerer Formen dieser Erkrankung. Dazu muss dem Fach sowohl ein höherer Stellenwert in der universitären Ausbildung zukommen als auch die postgraduale Ausdifferenzierung von Spezialisten oder Fachzahnärzten für Parodontologie deutschlandweit vorangetrieben werden. Die neue Behandlungsrichtlinie für die PAR-Therapie, die ab dem 01.07.2021 in deutschen Zahnarztpraxen wirksam wird, erlaubt die Versorgung parodontal erkrankter Patienten auf Basis international anerkannter wissenschaftlicher Standards und verbessert damit die Rahmenbedingungen für die Parodontitistherapie in der zahnärztlichen Praxis.

## Footnotes

1 https://www.g-ba.de/beschluesse/4623/.

2 BEMA: Bewertungsmaßstab zahnärztlicher Leistungen.

3 App-Version unter https://apps.apple.com/de/app/parodontitis-selbsttest/id504498921, Desktop-Version unter https://selbsttest.dgparo.de.
